# Jacob Disease, Osteochondroma of the Coronoid Process, Coronoid Process Hyperplasia or Langenbeck Disease: The Big Jumble. Comment on Raccampo et al. Jacob’s Disease: Case Series, Extensive Literature Review and Classification Proposal. *J. Clin. Med.* 2023, *12*, 938

**DOI:** 10.3390/jcm12154966

**Published:** 2023-07-28

**Authors:** Léa Mattei, Gwénaël Raoul, Matthias Schlund, Romain Nicot

**Affiliations:** 1 University Lille, CHU Lille, Department of Oral and Maxillofacial Surgery, 59000 Lille, France; 2 University Lille, CHU Lille, INSERM, Department of Oral and Maxillofacial Surgery, U1008-Advanced Drug Delivery Systems, 59000 Lille, France; 3 University Bordeaux, CHU Bordeaux, INSERM, Service de Chirurgie Maxillo-Faciale et Stomatologie, U1026-Bioengineering of Tissues, 33000 Bordeaux, France

We read the article by Raccampo et al., about Jacob disease and their ambiguous definition of the condition. 

In this article, the authors state that Jacob disease “is defined by most as the formation of a pseudojoint between the inner surface of the zygoma and the coronoid process of the mandible, which can be deformed or elongated by several pathological processes” [1].

It should be noted that in his postmortem description of joint formation between the zygomatic bone and the mandibular coronoid process, Octave Jacob described a coronoid process of normal shape and size associated with a hyperostosis of the posterior part of the zygomatic bone. The posterior surface of the zygomatic bone and the coronoid process were covered by cartilaginous tissue, forming a pseudojoint [2].

Therefore, the definition of Jacob disease should be limited to pseudojoint formation between the zygomatic bone and the coronoid process of the mandible without any assumptions about coronoid process condition. 

In addition, cases described in some of the cited articles can maintain a certain level of confusion about Jacob’s disease definition. Gibbons et al., described a case of coronoid process hyperplasia (CPH) with exostosis of the zygomatic bone, but the histopathology found no cartilaginous tissue and therefore no pseudojoint formation, which is mandatory to diagnose Jacob disease. Tavassol et al., also found an elongated coronoid process of the mandible “locking above the zygomatic arch”, but no pseudojoint formation was described on computed tomography or histopathology analysis. Sawada et al., reported a case of osteochondroma of the coronoid process of the mandible but underlined that “there was no pseudojoint formation between the zygoma and the lesion” [1].

It appears important to highlight three different conditions confused in the previous cases:

- Mandibular CPH, or Langenbeck disease, is characterized by an abnormally elongated coronoid process of the mandible, made of histologically normal bone tissue, responsible for impingement with the zygomatic bone [3].
- Osteochondroma of the coronoid process is a benign bony neoplasm coated by normal cartilaginous tissue. Only the affected bone is covered by cartilage [4].
- Jacob disease consists of pseudojoint formation between the zygomatic bone and the coronoid process of the mandible [2]. It can be associated with CPH or not [5].

Jacob disease can therefore be an evolution and complication of both CPH and osteochondroma of the coronoid process of the mandible as well as any condition that results in a chronic impingement between the coronoid process and the zygoma, e.g., an untreated displaced zygomatic fracture. However, this diagnosis cannot be made if pseudojoint formation with cartilaginous tissue on both the zygomatic bone and coronoid process is not present (Figure 1).

## Figures and Tables

**Figure 1:**
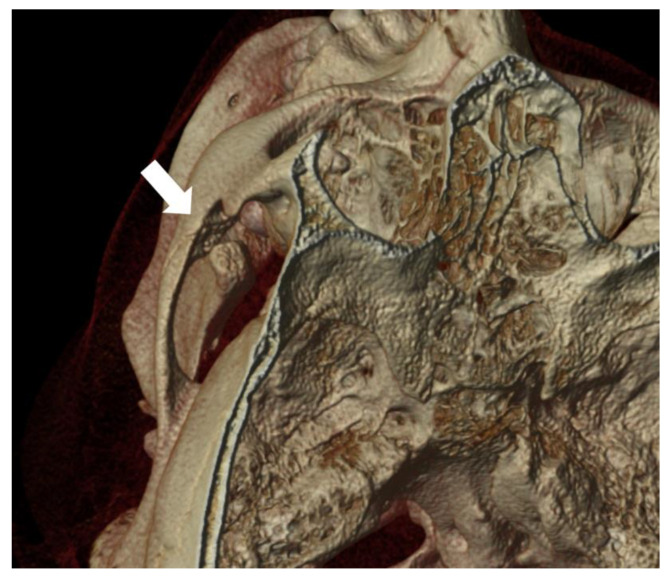
Three-dimensional reconstruction of computed tomography scan in a patient with Jacob disease. The white arrow shows the pseudojoint formation between the zygomatic bone and the elongated coronoid process of the mandible.

## References

[1] Raccampo L., Panozzo G., Tel A., Di Cosola M., Colapinto G., Trevisiol L., D’agostino A., Sembronio S., Robiony M. (2023). Jacob’s Disease: Case Series, Extensive Literature Review and Classification Proposal. J. Clin. Med..

[2] Jacob O. (1899). Une cause rare de constriction permanente des machoires. Bull. Mém. Soc. Anat. Paris.

[3] Mattei L., Raoul G., Barry F., Ferri J., Nicot R. (2023). Is panoramic radiography adequate for diagnosing coronoid process hyperplasia? A case series. J. Stomatol. Oral Maxillofac. Surg..

[4] Kerscher A., Piette E., Tideman H., Wu P. (1993). Osteochondroma of the coronoid process of the mandible. Oral Surg. Oral Med. Oral Pathol..

[5] Domart M., Nicot R., Mattei L., Cloître A., Lesclous P., Bertin H., Corre P. (2023). Effectiveness of treatment by coronoidectomy and active rehabilitation in Langenbeck or Jacob diseases. A retrospective study of 20 cases. J. Stomatol. Oral Maxillofac. Surg..

